# L2 Acquisition of a Complex Stress Pattern: UG-Constrained Learning Paths in Khalkha Mongolian

**DOI:** 10.3389/fpsyg.2021.627797

**Published:** 2021-08-24

**Authors:** Öner Özçelik

**Affiliations:** Department of Central Eurasian Studies, Indiana University, Bloomington, IN, United States

**Keywords:** stress, Mongolian, acquisition of prosody, learnability, UG, default-to-opposite edge stress, L2 acquisition of phonology

## Abstract

This paper examines second language (L2) acquisition of stress in Khalkha Mongolian, which is one of the few Default-to-Opposite Edge stress systems of the world, and as such, demonstrates “conflicting directionality” regarding stress assignment, resulting in the leftmost edge of a word being more prominent in certain words and the rightmost edge in certain others. Given the additional fact that the language exhibits Non-finality effects, and that, unlike English, codas are not moraic, its acquisition presents unique difficulties and challenges for English-speaking learners of the language. Many of these challenges potentially lead these learners to make Universal Grammar (UG)-unconstrained (but cognitively reasonable) assumptions about how the phonology of Mongolian works, especially since the learners do not have all the Mongolian data available to them all at once. The learning scenario here, thus, provides unique opportunities to investigate whether L2 phonologies are constrained by the options made available by UG. The findings of a semi-controlled production experiment indicate that although learners do not necessarily converge on the prosodic representations employed by native speakers of the L2 (i.e., footless intonational prominence, at least for the leftmost/default edge ‘stress’), and although certain changes to the grammar are very difficult to implement, such as switching from moraic codas to non-moraic codas, the learners nevertheless demonstrate a stage-like behavior where each step exhibits the parameter settings employed by a natural language, one that is neither like the L2 nor the L1. Conversely, despite the input leading them to do so, learners do not entertain UG-unconstrained prosodic representations, such as End-Rule-*Middle* or End-Rule-*Variable*; End-Rule is set either to *Right* or *Left*, as is expected in a system constrained by the options made available by UG. We conclude that the hypothesis space for interlanguage phonologies is determined by UG.

## Introduction

Languages demonstrating ‘conflicting directionality’ as concerns ‘stress’ provide phonologists with intriguing opportunities to investigate the options made available by Universal Grammar (UG), and have informed *all* major theories of stress (see e.g., Hayes, 1981, 1995; Prince, 1983; Halle and Vergnaud, 1987; Idsardi, 1992; Kenstowicz, 1995; Zoll, 1997, among others). Also called Default-to-Opposite Edge (DOE) stress systems, stress in these languages falls on a property closest to one edge of a word (say, the leftmost syllable demonstrating a certain property, e.g., long vowels), but if no such property is present within the word, the opposite edge of the word (e.g., the rightmost syllable) attracts stress. Highly researched in formal theoretical phonology, this pattern has so far not been investigated in second language (L2) acquisition research despite the unique insight it can offer to the research on learnability. One reason for this is the fact that very few people are second language speakers of these languages, which are solely composed of almost never taught languages, such as Buriat, Chuvash, Huasteco, Mari, Khalkha Mongolian, and Selkup.

In this paper, I investigate the L2 acquisition of one such language, Khalkha Mongolian, a language rarely taught outside of Mongolia, but is perhaps still the most commonly taught language among those demonstrating DOE stress, as others are never taught in the West, excluding occasional instruction on their structures, usually by a temporary visiting scholar. Further, it displays additional sources of complexity, not seen in most other DOE stress languages, such as the presence of Non-finality effects. Therefore, the current study contributes significantly to our understanding of L2 acquisition of stress, adding to a small but growing body of literature on the subject (see e.g., Archibald, 1992, 1993, 1995, 1998; Broselow and Park, 1995; Pater, 1997; Tremblay, 2008; Özçelik, 2011, 2016, 2018; Garcia, 2016, 2020).

In Khalkha Mongolian, the standard variety of Mongolian, the location of primary stress is determined by the following rule, provided in (1), which is itself composed by three parts, each of which is exemplified below in (2) to (4) with Light (L) and Heavy (H) syllable combinations:


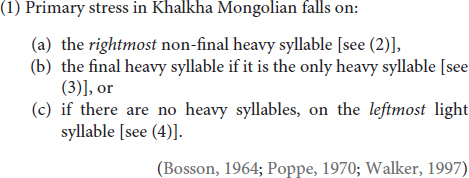


These three tenets of stress assignment in Khalkha are illustrated in (2) through (4) below, where Heavy (H) stands for a syllable that contains a long vowel, with Light (L) standing for a syllable with a short vowel, with or without a coda consonant (i.e., codas are not moraic in Mongolian and its various dialects).


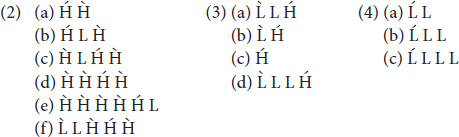


Given the additional Non-finality effect, illustrated in (2) [but compare with (3); see also (1a)], which most DOE stress languages do not entertain (see e.g., Gordon, 2000), this is a system even more complicated on the surface than other languages displaying DOE stress, making Khalkha one of the most complex regularly stressed languages in the world.

In order to see the challenges this learning scenario potentially poses for the learner in the absence of access to a domain-specific knowledge about the structural possibilities natural languages offer, consider the following: On the surface, it looks like Mongolian is a Trochaic and Weight-Sensitive language, but a strange one in that End-Rule, the parameter that determines the location of main stress, appears to be sometimes set to *Left* (2a, b), sometimes to *Right* (3a, b, d), and sometimes even to *Middle* (2c, d, f), and sometimes replaced by Leftmost-Wins (4c), a system that is linguistically impossible [more on this in (7) and (8) below], and crucially one that is, thus, ruled out by UG. If, however, these systems are considered to be prominence-driven, and independent of foot structure (Walker, 1997), or if the default (left edge) ‘stress’ at least were to be considered ‘intonational prominence’ instead of stress (e.g., Gordon, 2000; Özçelik, 2017), such systems would find a more viable explanation, especially since the Foot is no longer considered to be a universal, i.e., not every prosodic word needs to be headed by at least one foot (e.g., Özçelik, 2011, 2014, 2017; Garcia and Goad, 2021).

To make things even more complicated, acquiring target-like stress in Mongolian requires, for speakers of languages like English, moving from a system where the grammar is weight-sensitive both to the weight contributed by long vowels and by coda consonants to a system where weight sensitivity is only to long vowels. This is because, unlike English, codas are not moraic in Mongolian, while vowels are. This means that English-speaking learners of Mongolian will have to unlearn weight-sensitivity to codas. This is an interesting scenario, because although we know, based on previous research on the L2 acquisition of English stress, that acquiring a weight-sensitive L2 when the L1 is weight-insensitive is possible, there has been no research investigating the mirror image of this situation, one where L2 learners need to unlearn a property, e.g., the state of being weight-sensitive to codas. For example, Pater (1997) found that Quebec French-speaking learners of English can acquire weight sensitivity in L2 English, even though the L1 is weight insensitive. Further, distinguishing between weight sensitivity to the nucleus vs. codas, Archibald (1993) demonstrated that learners whose L1 is sensitive to the weight of the nucleus (i.e., where long vowels contribute weight) can switch to a system like English, where the language is weight-sensitive not only to the nucleus, but also to the rhyme (i.e., codas, along with long vowels, are moraic). The opposite direction, one where learners need to move from a system where the L1 is weight-sensitive both to the nucleus and the rhyme (e.g., English) to one where the L2 is weight-sensitive only to the nucleus (e.g., Mongolian), a potentially more difficult acquisition scenario, is yet to be investigated to my knowledge.

As seen, Mongolian has a very complex stress system, and the acquisition task is expected to be rather difficult for its learners. To complicate things even further, even linguists do not concur regarding the exact location of stress in Mongolian. Svantesson et al. (2005), in their book, *The Phonology of Mongolian*, point this out, stating “there are widely differing opinions on the place and nature of word stress in Mongolian” (p. 96), exemplifying at least six different opinions in the literature on the place of stress in Mongolian. Nevertheless, Walker’s (1997) description has gained popularity in recent theoretical research, with her convincing arguments that much of the disagreement on the location of stress in Mongolian originates from a lack of understanding of Non-finality effects, which, in fact, are not possible to observe in Khalkha unless a word contains more than two heavy syllables [e.g., as in (2d–2f)]. Looking into most other word types, one will thus get the (incorrect) impression that Mongolian stresses the leftmost heavy syllable [as in (2a-2b) and (3)] and otherwise the leftmost light syllable [as in (4)], i.e., a system that would put Khalkha among Default-to-*Same* Edge languages (as opposed to DOE), unless, of course, one looks into forms that contain more than two heavy syllables [as in (2c–f)], combinations that naturally form a small subset of words in Mongolian (or any language). It is perhaps for this reason, as Walker (1997) emphasizes, that many of the earlier descriptions of Mongolian stress have later been found to be incorrect, with certain authors updating their own analyses in their later research. For example, Poppe (1951) presented Mongolian as a Default-to-Same Edge system, updating it later in Poppe (1970) into an argument more in line with a DOE system (Walker, 1997).

Given all these issues, it should come as no surprise that the L2 acquisition task should be extremely difficult and confusing (see below) for learners of Mongolian, especially given the fact that the learners do not have *all* the Mongolian data available to them all at once. In fact, target-like representations may not *ever* be reached. Partially for this reason, however, and as the input is not very helpful in that it does not give unambiguous evidence as to the parameters of stress assignment in Khalkha, the acquisition task here presents us with intriguing opportunities to examine the various alternative constructions learners may indeed come up with, as well as various others they may not ever entertain.

In fact, I will demonstrate that this unique acquisition task presents strong evidence that L2 prosodic grammars are constrained by the options made available by UG (Broselow and Finer, 1991; Archibald, 1993; Goad and White, 2008, 2019; Özçelik, 2016, 2018), thereby offering support for UG-based approaches to L2 acquisition (White, 1989, White, 2003b; Schwartz and Sprouse, 1996). As will be made clear later, in restructuring their grammar, learners of Mongolian consider only UG-constrained options and do not entertain options that are not permitted by UG. Further, in doing so, a stage-like behavior emerges as learners continue to reset various prosodic parameters, a stage-like behavior that ultimately makes the grammar more target-like (at least on the surface). Before such target-like behavior emerges, however, various intermediary stages arise which are neither like the L1 nor like the L2, and are sometimes more unlike the L2 than the initial stage (the L1), both formally and with respect to the location of stressed syllables on the surface, a fact that finds no explanation based on input alone or L1 transfer alone.

In addition, this paper also sheds light on the issue of variability in interlanguage grammars, a topic that has recently generated much fruitful discussion in syntax and morphology, particularly with respect to variable omissions of functional morphology (see e.g., Lardiere, 1998a, b; Ionin and Wexler, 2003; White, 2003a; Ionin et al., 2004 for different accounts of variability), but has received almost no attention in phonology, even though successful phonological explanations have been offered to explain variability in morphology and syntax (see e.g., Goad et al., 2003; Goad and White, 2004, 2006, 2019). Explaining variability in phonology itself is crucial, because along with variability in suppliance of functional morphology, phonological variability in interlanguage grammars is perhaps the leading indication of non-native-like performance, as it is persistent even in end-state grammars (see e.g., Özçelik, 2016, 2018).

The remainder of the paper is organized in the following way: in Section “Representation of Stress: the L1–L2 Language Background,” we review a range of facts about stress and prominence in both Mongolian and English. This section also outlines our hypotheses. Section “Materials and Methods” then describes the design of the experiment that was employed to test these hypotheses and the participants who took part in the experiment. The results are then presented in Section “Results and Discussion” along with a discussion of their implications for UG and variability in L2 phonology. Finally, Section “Discussion and Conclusion” concludes the paper.

## Representation of Stress: The L1–L2 Language Background

### Mongolian Stress

The stress pattern of Khalkha Mongolian has already been described above in (1). As mentioned there, this pattern is potentially highly challenging for any learner, irrespective of the L1, making Mongolian one of the most challenging regularly stressed languages to acquire in the world. Unless one has a means of analyzing and comparing (almost) all Mongolian words all at once, reaching the correct generalizations, presented in (1) above, on the basis of primary linguistic data alone is then extremely challenging, a task that may never be accomplished by L2 learners. In fact, as has already been mentioned above, this stress pattern has been challenging even for linguists to correctly describe the rules of stress assignment in Mongolian (e.g., Svantesson et al., 2005)^1^.

Further, acoustic correlates of stress/prominence are also somewhat different in Khalkha Mongolian than in English, perhaps with the partial exception of duration, thereby potentially adding to the challenges of learning such a system. Although there has not been much research on the acoustics of stress/prominence in Mongolian, two studies give us some insight into the issue: Harnud (2003) and Sang and Martin (2012), the former on Inner Mongolian spoken in China and the latter on the standard Khalkha variety spoken in Mongolia. As the Inner Mongolian variety appears to behave somewhat differently with regard to the location of stressed syllables from Khaklha, and as Khalkha is the variety under investigation here, we will focus on Sang and Martin’s analysis, which examined F0 and duration only with bisyllabic and trisyllabic stimuli [various aspects of which (e.g., Non-finality) were not controlled, nevertheless providing an overall picture of the phenomenon]: For words with only light syllables [i.e., those in (4)], they found that the first syllable (which corresponds to the stressed syllable) consistently had the lowest F0 value (as opposed to English words where the stressed syllable bears the highest F0). For words with at least one heavy syllable, on the other hand, the heavy syllable consistently had the highest F0 value. When the word had multiple heavy syllables, the leftmost heavy syllable usually had the highest F0, whereas in some cases the rightmost syllable did. As for duration, for words containing only short vowels, the duration of the first vowel was always greater (1.5 times) than the duration of the other vowels, somewhat mirroring English stress which is also accompanied by greater duration, though to a much smaller extent. For words containing a single long vowel, the long vowel was much longer (2.4 times) than the short vowels. And for words containing multiple long vowels, the leftmost long vowel was on average longer (1.3 times) than the other long vowels. Finally, Sang & Martin do not provide values for intensity; however, Harnud (2003) reports, for Inner Mongolian, that for words with first short vowel the second syllable bears greater intensity, while for words with first long vowel, the first syllable bears greater intensity. In this latter scenario, the second syllable bears greater F0, thereby creating a context where intensity and F0 are contradict, at least for Inner Mongolian.

To exacerbate this already arduous task of simply ‘describing’ the Mongolian stress pattern in layman words, accounting for it or even simply defining it with the parameters of stress assignment (see e.g., Dresher and Kaye, 1990; Hayes, 1995) usually leads to difficulties that, on the surface, appear to predict a UG-unconstrained language, as will be explained below. Still, with respect to certain parameters at least, things look straightforward: Regarding the parameter Foot-Type, for example, Khalkha appears, on the surface, to be a Trochaic (head-initial) language, given initial stress in forms like (4). In addition, given the fact that heavy syllables attract stress when available, as with the forms in (2) and especially (3) (where all Hs bear primary stress), it looks clear that Weight-Sensitivity is set to *Yes*. Further glance at the data reveals some complications however; for example, End-Rule, which determines the location of primary stress, appears to be sometimes set to *Left* (1a,b), sometimes to *Right* (2a,b,d), and sometimes even to *Middle* (1c,d,f), as evidenced by the fact that when there are multiple stresses available in a given word, it is sometimes the leftmost, sometimes the rightmost and sometimes the middle one that bears primary stress, indicated here with an acute accent. Furthermore, sometimes, it looks like End-Rule is replaced by Leftmost-Wins (i.e., out of multiple possible stresses, only the leftmost one arises, instead of making one the most prominent but still keeping the others as secondary).^2^ In other words, on the surface, this looks like a system that is linguistically impossible and crucially one that is ruled out by the options made available by UG.

These problems, however, go away if initial (leftmost) stress in DOE stress languages like Khalkha is assumed to be intonational prominence, as with Gordon (2000, 2014) and Özçelik (2014, 2017), one that does not involve foot structure. This would mean that End-Rule is consistently set to *Right* in Mongolian (with final foot extrametricality), and End-Rule-*Right* is vacuously satisfied for cases with initial stress, as there is no foot available. This is an analysis that is supported by both acoustic data (Gordon, 2000; Svantesson et al., 2005) and typological considerations regarding stress systems (Özçelik, 2014, 2017), but is still one that is very difficult to reach on the basis of the input alone, the primary linguistic data available to the learner (see section “L2 Acquisition of Mongolian Stress” below).

### English Stress

English, the first language of the learners tested in the current study, also has a very complex, but at the same time, a very well-defined stress system, one that differs significantly from that of Mongolian. In English, every (lexical) word is assumed to contain at least one foot (see e.g., Liberman and Prince, 1977; Hayes, 1981, 1995; Halle and Vergnaud, 1987), as evidenced by the fact that every prosodic word (PWd) has at least one stressed syllable in English, and that there are no words smaller than a binary foot; therefore, syllables that form a word on their own either contain a long vowel (e.g., /zu:/ ‘zoo’) or end in a closed syllable (e.g., /kæt/ ‘cat’), with no word types such as /zu/ and /kæ/, meaning that words are composed of a minimum of a binary foot, one that is binary at the moraic level. Further, both long vowels and nucleus vowel + coda consonant sequences are bimoraic in English, as both vowels and codas are moraic in this language.

The complex stress system of English can easily be summarized referring to various parameter settings that all have to do with the way syllables are constructed into feet, as follows:


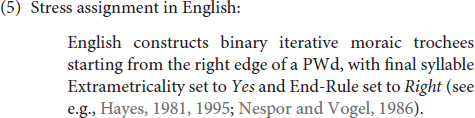


What this means will be explained in detail below, where we cover each relevant parameter one by one, i.e., Extrametricality, Directionality, Foot Binarity, Foot Type, Iterativity, End-Rule and Weight Sensitivity respectively. This discussion will help the reader better understand the results of the study later, since, as will be demonstrated in the Results section, English speakers gradually move away from the English settings of these parameters [see (6) through (12)] in creating interlanguage representations. In demonstrating these parameter settings, we focus on one example, the word *originality*, and illustrate how, given all the parameters of stress assignment, syllables are constructed into feet in English. First, examine (6a), which illustrates that the parameter Extrametricality is set to *Yes* in English, which means that all word-final syllables are ‘ignored’ as far as stress assignment (or rather foot construction) is concerned.^3^


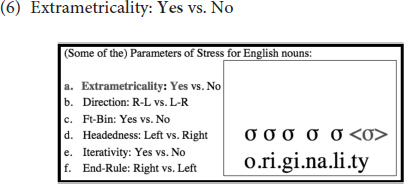


After the final (extrametrical) syllable is skipped [see (6)], foot construction starts at the right edge of the word, as illustrated in (7), since the Directionality parameter is set to *Right-to-Left* in English (as opposed to the alternative *Left-to-Right*):


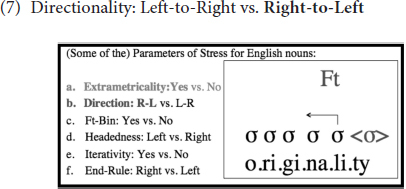


Furthermore, as demonstrated in (8), as the Foot Binarity (Ft-Bin) parameter is set to *Yes*, all feet are binary in English (as with the great majority of the world’s languages, as this setting is usually viewed to be near-universal, see e.g., Hayes, 1995). This means that each foot must be composed of two syllables (or moras – see below) in English:


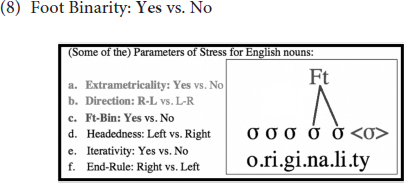


(9) below indicates, in addition, that the Foot-Type/Headedness parameter is set to *Left* in English, meaning that feet are left-headed, and thus, trochaic (instead of being right-headed/iambic), as the leftmost syllable within the foot is the one that bears the greatest prominence (and is, as such, called the head):


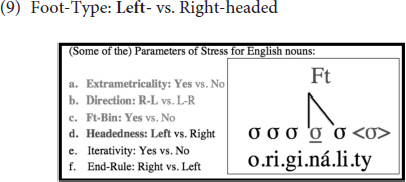


Further, as illustrated in (10), if a word is long enough to accommodate multiple binary feet, multiple feet can then be created in English, i.e., instead of leaving the remaining syllables unfooted, as some languages would do. That is, footing in English is iterative, and thus, in words that are long enough, multiple stresses emerge, suggesting that the Iterativity parameter is set to *Yes* in English:


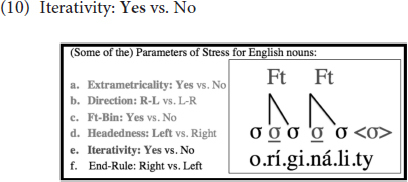


Finally, note that when there are multiple syllables that bear stress in a given word, it is the rightmost stressed syllable that is elevated to function as primary stress, and others are demoted to bear the secondary stress status, as indicated in (11) below. This means that End-Rule is set to *Right* in English, instead of *Left*.


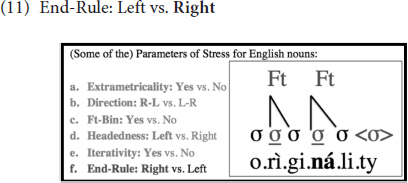


One parameter that is not illustrated above with the word ‘originality’ but is nevertheless important for the discussion in this paper is Weight-Sensitivity, which is set to *Yes* in English, and as such, heavy syllables, whether they are heavy because they contain a long vowel or a coda consonant, bear stress. Feet are, thus, binary at the moraic level in English. This is clarified by means of a comparison of two words in (12); whereas the first syllable is stressed in the first one, the second syllable is stressed in the second. This is because the second syllable in the second word can create a binary foot of its own since it has two moras, one of which comes from the vowel and the other from the coda consonant:


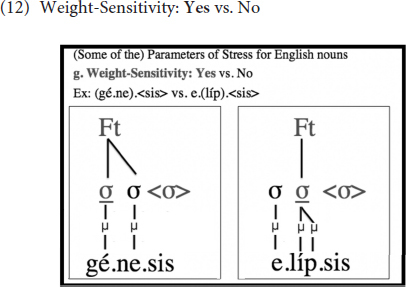


### L2 Acquisition of Mongolian Stress

#### The Learning Challenge

As has been established above, the word prosodic grammars of Mongolian and English differ significantly. In acquiring the target language and going through the process of restructuring their grammars, English-speaking learners of Mongolian need to make several significant changes in various parameter settings. First of all, if, as we have illustrated above, the default initial prominence in Mongolian is footless intonational prominence rather than (footed) stress, English-speaking learners will eventually need to have footless representations, at least for words that contain no heavy syllables (i.e., initial default stress). However, since representing footless words in an L2 is an extremely challenging task for learners with footed L1s (Özçelik, 2011, 2016, 2018) and assuming thus that English-speaking learners of Khalkha will always produce footed outputs, in order to accommodate the L2 input with a footed grammar, one could claim that Khalkha L2ers will possibly be forced to make several UG-unconstrained assumptions about the language, where End-Rule is sometimes set to *Right*, sometimes to *Left* and, even more surprisingly, sometimes to *Middle*, as has already been mentioned in Section “Introduction” and illustrated in (13) below. In fact, for words that that are long enough and only contain a light syllable, Leftmost-Wins, one could say (though see below), will replace End-Rule [see (14)]:


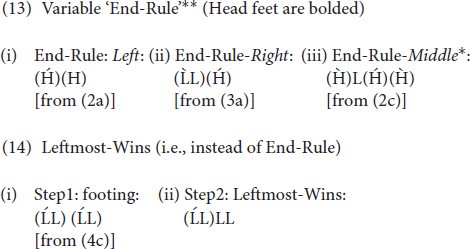


If interlanguage grammars are constrained by UG, however, as I hypothesize they are, learners will not make such assumptions; rather, they will produce words that are consistent with either End-Rule-*Left* or End-Rule-*Right* only, despite the input and despite the fact that neither may capture the full array of primary linguistic data in Mongolian.

Even if we assumed initial default prominence in Khalkha to be trochaic (i.e., foot-based), English-speaking learners of the language will face similar challenges, except for having to expunge the Foot, of course. In other words, assuming that they may not ever be able to expunge the Foot (Özçelik, 2016, 2018), whether one assumes initial prominence to be trochaic or (footless) intonational prominence, they will, in the end, have to make similar rearrangements (which all involve the Foot) in parameter settings in consistently stressing the initial syllables of words composed only of light syllables. More specifically, learners will need to create either a binary trochee that is constructed from the left edge (i.e., with left-to-right foot construction) or an unbounded weight-insensitive trochee that encompasses the whole word, as illustrated respectively in (15a) and (15b) below. As (15a) is the more unmarked one of the two and almost all cases that refer to (15b) can be explained through (15a) (Hayes, 1995), I will, for the purpose of this paper, assume that this is the structure that they will eventually come up with, the structure that will make their interlanguage grammar most similar, at least on the surface, to that of the target grammar:


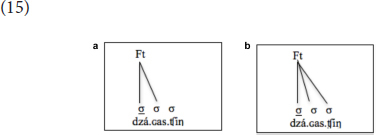


Another challenge for English-speaking learners arises from the fact that codas are moraic in English, as illustrated in (12) above; therefore, when a closed syllable is available, it is stressed, as with /e.líp.sis/ (compare with the initial stress in/gé.ne.sis/), just as syllables with long vowels are stressed, as with /a.ró:.ma/, since the language is weight-sensitive. In Mongolian, however, only long vowels attract stress; closed syllables do not, as codas are not moraic. As such, English-speaking learners of Mongolian will have to learn that codas are not moraic in Mongolian; otherwise, in Mongolian words that are composed of all short vowels, such as /dza.ɢas.ʧìŋ/, they would stress the closed syllables, even though these are light in Mongolian, since they are heavy in English. As the (un)learning of coda moraicity requires moving from a superset to a subset grammar, I predict that, unlike the opposite direction (see Archibald, 1993, 1998; Pater, 1997), this will be rather hard to acquire.

#### Hypotheses

I hypothesize that whether Khalkha initial prominence, the main focus of this paper, is formally footless or not, English-speaking learners of the language will always have footed representations, as expunging the foot, I assume, is impossible, once it is projected in the L1 (Özçelik, 2011, 2018). Further, given the Prosodic Acquisition Path Hypothesis (PAPH) (Özçelik, 2016) [see also the Full Transfer/Full Access Hypothesis (FTFA), Schwartz and Sprouse, 1996], in initial stages, they will construct feet as in English, that is, right-to-left iterative weight-sensitive trochees, with Extrametricality and Weight-Sensitivity both set to *Yes* and with moraic codas [see (6) and (12)], even though this will not, on the surface, consistently stress the correct syllables in the target language. Further, given the input, which is mostly composed of words that are word-initially prominent (i.e., words composed of all short syllables) or words prominent on the first long syllable (i.e., words composed of up to two long syllables), I hypothesize that the learners will gradually reset various parameters in the target language and in the end have left-to-right non-iterative (and weight-sensitive) trochees at advanced levels, as illustrated in (15a) above.

Given the PAPH, in resetting foot-related parameters, I hypothesize that the learners will go through various stages that correspond to bundles of different parameter settings, and as such, create grammars (and surface outputs) that differ from both the target language and the native language but are constrained by the options made available by UG.

I also predict them *not* to entertain certain options: (i) options that do not get triggered by input or serve to make at least some aspects of learners’ outputs more target-like, and (ii) options that would indeed make the grammar more target-like on the surface given the input, but are not permitted by the universal inventory of foot types. As per (i), I hypothesize, for example, that options that employ right-headed feet (iambs) will not be used, as these will not account for the initial prominence in words composed of all light syllables (default ‘stress’), nor will employing such an option increase their surface performance on words with heavy syllables: An iambic analysis of the former is simply not possible (unless one assumes rampant empty onset-nucleus sequences for all words starting with CV), and an iambic analysis of the latter is not superior to a trochaic analysis, as heavy syllables can be the head in either analysis, especially when feet are not rhythmic as with Mongolian. Finally, as per (ii), such options as ^∗^End-Rule-*Middle* will be ruled out, even though a learner that is driven by domain general cognitive principles, should presumably be able to favor such an option, especially given the input. This is because prosodic parameters refer to edges, either the rightmost or the leftmost edge being the head. Likewise, End-Rule should be set either to the *Right* or to the *Left*, but not to both, although, presumably, given that parameter resetting is possible, for some learners, both options may be at use for a period of time, while the grammar is still going through change. Still, it is not expected to observe many learners whose outputs are equally consistent with both the left and the right setting of prosodic parameters.

In order to test these hypotheses, an experiment was conducted involving words with various syllable structure profiles, which is the subject of the next section.

## Materials and Methods

In order to investigate the hypotheses laid out above, a semi-controlled production experiment was conducted with 12 L1 English-speaking learners of L2 Khalkha Mongolian, of various proficiency levels. Proficiency level was determined by means of two independent proficiency tests, the results of which closely matched self-report: (i) a cloze test to measure syntactic, morphological and discourse proficiency, and (ii) a read-aloud task to assess participants’ global phonological proficiency (see Akita, 2006, 2007 for a similar procedure and for more on the design and implementation of the read-aloud task). Level of proficiency was not used as a factor in recruiting participants, because the potential pool was very limited to begin with, and as such, the experiment was open to any (near-)monolingual English-speaking learner of Mongolian. As was determined by the results of the two proficiency tests, however, there were two novice (low beginner), three beginner, five intermediate, and two advanced learners.

The participants ranged in age from 20 to 34 years old. They started learning Mongolian either in college or in graduate school, and in all cases, after age 20. All of the participants had college education (or higher), or were, at the time, attending college or graduate school.

The stimuli included a total of 240 polysyllabic words, mostly nouns, of various lengths and syllable structure profiles. In some cases, nominals with various endings such as case markings, were also used, especially in an effort to find longer words (see Table 1). Verbs were avoided as they are targeted by slightly differently in English as a class. Further, for bisyllabic and trisyllabic stimuli, all possible Heavy (H) and Light (L) syllable combinations were represented. Although this was not always possible to do for longer words (e.g., with four or five syllables), various combinations of H and L were represented for these words, too.

**TABLE 1:** Example stimuli: words with multiple heavy syllables.

HH	HHL	LHH	HLH	HHH	HHHH
tuulaj	giiguulegch	ugaaltuur	böörönhii	aawaasaa	eejuudeeree
туулай	гийгүүлэгч	угаалтуур	бөөрөнхий	ааваасаа	ээжүүдээрээ
Rabbit	Consonant	Sink	Circle	From dad	By the mothers

Words with multiple Hs (60 in total) and words composed only of light syllables but with various combinations of open vs. closed syllables (60 in total) were the focus of this paper (in addition to 120 fillers). For the former, the following forms were analyzed: HH, HHL, LHH, HLH, HHH, and HHHH. There were 10 of each of these. Examples are provided below:

The first four of these will give us insight into whether End-Rule is set to *Left* or *Right* in the learners’ grammars, or to both, whereas the latter two (stimuli with more than two Hs) will additionally be informative as to whether learners employ the unattested End-Rule-*Middle* setting. The issue is complicated, however, by the previously unforeseen finding that in forms that contain a light syllable (e.g., HHL, LHH, HLH), if the light syllable is closed (i.e., has a coda consonant), as with the HHL example in Table 1, it was often treated as heavy by many English-speaking learners of Mongolian (see below for more on this), although closed syllables do not contribute weight in Mongolian (and should, thus, not normally be heavy or stressed). As such, the analysis here had to focus only on the following word types, in order to disentangle the two variables (target weight and weight incorrectly assigned by the learners): HH, HHH, and HHHH, which are all word forms where all syllables are heavy (with a long vowel or glide).

For words composed of only light syllables, the second main focus of this paper, coda profiles were controlled for all bisyllabic and trisyllabic stimuli, meaning that these represented all possible combinations of open (O) and closed (C) syllables, i.e., O.O, O.C, C.O, C.C for bisyllabic stimuli and O.O.O, O.O.C, O.C.O, O.C.C, C.O.O, C.O.C, C.C.O, C.C.C for trisyllabic words. There were five words within each subtype, amounting to 20 bisyllabic and 40 trisyllabic words with all light syllables. Table 2 below provides an example of each subtype for words with all light syllables:

**TABLE 2:** Example stimuli: words with all light syllables.

OO	OC	CO	CC				
**Bisyllabic:**							
buga	uzeg	taksi	devter				
буга	үзэг	такси	дэвтэр				
deer	pen	taxi	notebook				

**OOO**	**OOC**	**OCO**	**OCC**	**COO**	**COC**	**CCO**	**CCC**

**Trisyllabic:**							
ajaga	zahidal	lavanda	öchigdör	halbaga	urgamal	salfetka	sarmagchin
аяга	захидал	лаванда	өчигдөр	халбага	ургамал	салϕетка	сармагчин
cup	letter	lavander	yesterday	spoon	plant	napkin	monkey

As differing mechanisms of syllabification across the two languages could potentially confound the results, it was ensured, in preparing the stimuli, that all coda + onset sequences were either sonorant + obstruent, sonorant + sonorant, or obstruent + obstruent. This guaranteed that codas in Mongolian would be syllabified as codas in English, too instead of being syllabified as the first member of a following word-internal onset cluster, thereby avoiding the effects of a possible confounding variable, i.e., transfer of L1 syllabification strategies from English.

Each stimulus was presented in a carrier sentence, exemplified below in (16):


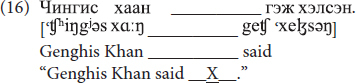


The learners’ task was to utter the entire carrier sentence. Before uttering stimuli in carrier sentences, however, the participants first had to utter them twice in isolation. Only the words produced in carrier sentences were later transcribed and analyzed. Words uttered in isolation were not analyzed, because these are problematic as various confounding variables could be involved, such as utterance-final lengthening, which occurs frequently across languages when a word is pronounced in isolation. In addition, word stress and phrase-level prominence are confounded when a word is uttered in isolation (Gordon, 2014; Hyman, 2014).

Praat acoustic analysis software (Boersma and Weenink, 2011) was used to analyze and transcribe the stimuli produced within the carrier sentences. In determining the presence and location of stressed syllables in experimental words, impressionistic data were used, but with back-up from spectrogram and waveform analysis. The following acoustic correlates were noted for each syllable in each stimulus: vowel and syllable duration (in ms), average and peak intensity (in dB), average fundamental frequency (F0, in Hz), and time of F0 peak. Further, as argued for by Peterson and Lehiste (1960), both spectrogram and waveform cues were employed for segmentation.

Each participant was tested individually in a sound-attenuated booth and was audiorecorded onto a computer using the Audacity software, and with the help of an external microphone. The following order of testing was employed: (i) a language background questionnaire, (ii) production experiment, and (iii) proficiency tests (the cloze test and the read-aloud task). The whole procedure, including the stimuli not to be covered in this paper, took about 1–1.5 h per participant.

## Results and Discussion

### General Observations and Variability

Before providing an in-depth formal analysis of the results, two general observations must be outlined, which were both immediately apparent at a first glance from the data collected. The first one involves variability *within* the outputs of the same learner and the second *across* different learners, both of which, I propose, can be captured in principled ways through L1 transfer and access to UG (more on this later). The two general observations are summarized in (17) below:


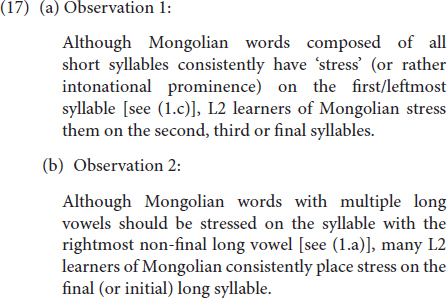


Notice that while one of these observations, i.e., Observation 1, seems to involve variability *within* individual grammars in that a given learner could pronounce three-syllable long target words like [Ĺ L L] sometimes as [L Ĺ L], sometimes as [L L Ĺ], and sometimes correctly as [Ĺ L L], such is not the case for Observation 2, which involves variation only *across* learners: That is, individual learners produce words with multiple Hs either with stress on the rightmost H or on the leftmost, but not sometimes on the leftmost and sometimes on the rightmost or ever on the one in the middle [even though this final strategy would capture many cases with multiple Hs, such as when there are three Hs in a given word, as in (2c)].

Below, detailed results are provided regarding the two observations, along with an explanation for these findings, followed by an in-depth analysis of individual results and individual learner grammars. Statistical analyses were not performed given the small sample (and population) size available and given that individual learner grammars were the focus. After all, the large number of options/parameter setting combinations that can lead to the same surface results (e.g., final vs. initial stress) effectively renders only individual results meaningful. We start with Observation 2.

### Observation 2 Explained: End-Rule Right or Left, but Not Both or Middle

Although six different word forms are provided in Table 1 above that could potentially be used to test the setting employed by the learners for End-Rule, only three of these were helpful in doing so, as has been mentioned above. This is because the learners often treated certain light syllables as heavy (more on this later), meaning that unless the word was composed of all heavy syllables in the target grammar, as with the forms HH, HHH, and HHHH, it was difficult to ascertain how many heavy syllables it had in the interlanguage grammar, potentially confounding the results. As such, and in order to avoid such a confound, the analysis here was restricted to the forms HH, HHH, and HHHH.

The analysis of these three word forms led to the findings illustrated in Table 3, for *each* of the 12 learners tested. For each word type, data indicate whether the leftmost, rightmost, or where relevant, the middle syllable was stressed, and to what extent.

**TABLE 3:** Results: location of main stress in percentage: rightmost, leftmost, or middle syllable within the word.

Subj.	HH		HHH			HHHH			Direct.
	Left	Right	Left	Mid	Right	Left	Mid	Right	
**Novice**									
M.I.	0	100	10	0	90	12.5	0	87.5	*Right*
A.B.	0	100	0	11.11	88.89	10	10	90	*Right*
**Beginner**									
T.H.	10	90	0	0	100	0	0	100	*Right*
P.L	0	100	10	0	100	10	0	100	*Right*
A.F.	80	20	77.78	0	22.22	75	12.5	12.5	*Left*
**Intermediate**									
K.G.	0	100	0	0	100	0	10	100	*Right*
D.B.	90	10	90	0	10	88.89	0	11.11	*Left*
B.B.	100	0	100	0	0	90	0	10	*Left*
J.K.	90	10	80	10	10	75	12.5	12.5	*Left*
E.S.	100	0	100	0	0	100	0	0	*Left*
**Advanced**									
K.K.	100	0	100	0	0	90	0	10	*Left*
K.H.	0	100	90	10	0	80	10	10	*Left?*

These results demonstrate that although some learners stress the rightmost syllable and some the leftmost, for each of the learners, *either* the rightmost syllable is consistently stressed, *or* the leftmost, and irrespective of whether the word has two, three, or four heavy syllables. In other words, learners do not stress the leftmost and rightmost syllables to an equal extent. These results indicate in no unclear ways that, for each learner, End-Rule was set either strictly to *Right* or strictly to *Left*. It was not set to *Middle* for any of the learners, nor was it ‘variable’ (i.e., sometimes *Left*, sometimes *Right*) in any of the interlanguage grammars involved, although one learner, one of the two most advanced, i.e., K.H., appears to constitute a partial exception to this pattern in that she has rightmost main stress for bisyllabic forms and leftmost for longer words.^4^ Although, for some of the learners, the syllable in the middle was, at times, promoted to the primary stress status, and although, for some, both left and rightmost syllables were occasionally stressed, this was likely performance-related, as the rate of occurrence for these phenomena is rather minimal.

It should be noted that these results (along with Observation 2) can be accounted for in a straightforward manner under the assumption that the options L2 learners entertain are constrained by the options made available by UG. As was mentioned in the introduction section, End-Rule can, after all, be set to either *Right* or *Left* in natural languages, but never to *Middle*, nor can it be set variably such that the same grammar gives outputs that are in line with both the *Right* and the *Left* setting of End-Rule (with the exception of grammar change/parameter resetting in progress). Such options are ruled out by UG. Given this, then, it is understandable why the learners tested here do not seem to employ a variable setting for End-Rule, one that fluctuates between End-Rule-*Right* and End-Rule-*Left*, and even End-Rule-*Middle* at times, although this would have been perfectly compatible with the primary linguistic data that they receive (and is a cognitively reasonable strategy, though linguistically incorrect).

It should also be noted, as one reviewer points out, that the final syllable receiving stress here for many learners, despite English opting for the *Yes* setting of the Extrametricality parameter, is still something that can be explained through English, the L1, as these are composed of final long vowels which are not extrametrical in English, unlike final closed syllables (see e.g., Hayes, 1982), as will be shown below. Although this is true, and may also explain, to a great extent, the dichotomy observed in the learners’ outputs as concerns extrametricality in words ending in syllables with long vowels vs. those ending in codas, it does not, by itself, explain why the initial syllable sometimes gets stressed and why the middle syllable never gets stressed, despite the primary linguistic data and despite the fact that English stresses the word-medial syllables, too, at least when the final syllable is heavy by means of ending in a coda consonant, and most subjects in this study still treated such forms as heavy, as will be explained later.

As mentioned above, variability in the setting of this parameter was observed only *across* learners, with exactly half of learners consistently employing the End-Rule-*Right* setting of this parameter (as with L1 English) and the other half the End-Rule-*Left* setting (with a few exceptions in a handful of words). Notice that neither setting alone gives target-like results 100% of the time, but the *Left* setting results in correct results for a greater number of cases than the *Right* setting (especially in cases with all light syllables or up to two heavy syllables), although, as explained in Section “Introduction,” formally speaking, the *Right* setting might be the correct one (with Non-finality taken into account). In a sense, then, formally speaking, beginners, who just employed the L1 setting of this parameter with no further change, were more correct with respect to the correct setting of this parameter than most of the other learners, but less correct in capturing the correct location of stress on the surface (because of Non-finality).

### Observation 1 Explained: Weight-Sensitivity Is Still Set to Yes

Turning back to Observation 1, for which we will provide a more detailed analysis, an examination of the results on stimuli composed of all light syllables, i.e., words that are consistently stressed on their first syllable in native Mongolian, confirms that there was a great amount of intra-learner variability regarding the location of stressed syllables. Unlike the situation with Observation 2 (see above), this variability was present in the outputs of the *same* learners. In other words, there were learners who sometimes stressed the first, sometimes the second and sometimes the final syllable of trisyllabic stimuli composed of all light syllables. These results are summarized in Table 4 based on proficiency level and the location of the syllable stressed, for both bisyllabic and trisyllabic stimuli.

**TABLE 4:** General results on stimuli with all light syllables: percentage stressed.

	Bisyllabic		Trisyllabic		
L1 Eng. (*n* = 12)	Penult	Final	Antepenult	Penult	Final
Novice (*n* = 2)	89	10.28	48.61	47.37	4.02
Beginner (*n* = 3)	36.18	63.82	26.06	30.89	43.04
Intermediate (*n* = 5)	49.53	50.47	57	27	16
Advanced (*n* = 2)	92.5	7.5	93.75	3.75	2.5

As seen, except for the two advanced subjects, who were able to consistently stress the word-initial syllable in both bisyllabic and trisyllabic words, the results do not appear to follow any specific pattern. To give an example, all three syllables of trisyllabic stimuli are stressed roughly to the same extent by the beginners, although the first syllable is consistently stressed by the novice learners for bisyllabic stimuli. In contrast, for the intermediates, the first and the second syllable of bisyllabic words are stressed equally frequently. Closer investigation of these results indicate, however, that there is, in fact, a pattern that lies behind this apparent disarray, one that is shadowed by the fact that providing results as generally as is done in Table 4 collapses different types of behavior under ‘proficiency levels,’ which are categorical variables that are non-linguistic. As such, individual results are more meaningful, as illustrated below in Table 5.

**TABLE 5:** Individual results on stimuli with all light syllables: percentage stressed.

	Bisyllabic		Trisyllabic		
	σ1	σ2	σ1	σ2	σ3
**Novice**					
M.I. *Stage 1^5^*	94.44	5.56	47.22	50	2.78
A.B. *Stage 1*	84.21	15.79	50	44.74	5.26
**Beginner**					
T.H. *Stage 2*	22.22	77.78	5.56	33.33	61.11
P.L *Stage 2*	26.32	73.68	2.63	36.84	60.53
A.F. *Stage 4*	60	40	70	22.5	7.5
**Intermediate**					
K.G. *Stage 2*	40	60	7.5	27.5	65
D.B. *Stage 3*	55	45	57.5	35	7.5
B.B. *Stage 4*	55	45	72.5	22.5	5
J.K. *Stage 4*	52.63	47.37	80	20	0
E.S. *Stage 4*	45	55	67.5	30	2.5
**Advanced**					
K.K. *Stage 5*	90	10	92.5	5	2.5
K.H. *Stage ?*	95	5	95	2.5	2.5

*^5^The stages (Stage 1, 2, etc.) cited here will be more meaningful in the next section, when this will be illustrated for each subject, along with an explanation of each percentage value.*

These individual results indicate that some learners behave very much alike, and these learners can thus be categorized together based on similar ‘linguistic behavior.’ For example, novice learners never stress final syllables, but for trisyllabic stimuli, they stress initial and penultimate syllables to the same extent. Many (but not all) of the intermediates stress initial and final syllables of bisyllabic stimuli roughly to the same extent, but when it comes to trisyllabic stimuli, stress usually falls on the initial syllable, with fewer words stressed on the penultimate syllable and almost no words on the final syllable.

I will demonstrate that patterns like these, as well as the apparent variability, can be captured by having recourse to the options made available by UG. Although this variability is observed not across learners but in the outputs of the same learners, it is, nevertheless, ultimately principled. While a given English-speaking learner of Mongolian may stress the first, second or third syllable of, for example, a trisyllabic word in Mongolian that is composed of all short vowels, as I will illustrate below, there is systematicity in choosing the syllable that is to be stressed, and the location of stress is, thus, predictable.

Regarding the systematicity, both parameters of UG and language transfer play a role, as with Observation 2 above, although the complex interaction between UG parameters and language transfer leads to surface variability in the utterances of the same learners in this case, variability that can be explained with recourse to (the changes made in the settings of) prosodic parameters.

More specifically, the underlying reason for Observation 1 to emerge is, I propose, the fact that English-speaking learners of Mongolian (especially those at beginning levels) still analyze closed syllables as heavy (H), even though only syllables with long vowels can be H in Mongolian, as opposed to English in which both long vowels and codas are moraic, meaning that closed syllables, as with syllables containing a long vowel, can be H.

Since, in the interlanguages of the learners, these stimuli are thus composed of a number of H and L syllable combinations, and assuming that English-speaking subjects can still make a variety of changes to the settings of the prosodic parameters exemplified in Section “English Stress” above (e.g., Extrametricality, Headedness, etc.), along with the Full Transfer/Full Access Hypothesis (Schwartz and Sprouse, 1994; 1996), both intra- and inter-learner variability in location of stress are naturally accounted for. In fact, as I will demonstrate below, a stage-like behavior emerges as learners of Mongolian make a variety of changes to the settings of prosodic parameters, while partially continuing to transfer from the L1 at the same time. This also results in a variety of grammars that are neither like the L1 nor like the L2, and can, thus, not be explained with input alone or transfer alone, i.e., without referring to the principles of UG.

### Prosodic Parameter Resetting: Stage-Like Prosodic Acquisition

We will illustrate the aforementioned stage-like behavior by examining individual learner grammars and by categorizing these grammars based on what parameters the subjects reset and how many of them. In doing so, we will focus on stimuli that are composed only of syllables with short vowels, which were created to accommodate all possible combinations of open and closed syllable types (see Table 2 in section “Materials and Methods”). As it is impossible to cover all of these words here within the space allotted, we will focus on three trisyllabic words, (i) /dzá.Gas.ʧiŋ/ <загасчин> “fisher,” (ii) /úr.Ga.mal/ <ургамал> “plant,” and (iii) /bá.ga.nə/ <багана> “column.” These respectively represent LHH, HLH, and LLL syllable structure types from the perspective of the English grammar (all LLL in Mongolian), as codas, as stated above, are moraic in English, unlike in Mongolian.

When we look at the outputs of individual learners with respect to these word types, a stage-like performance pattern emerges, with some learners behaving more on the L1 English side of the spectrum with respect to various parameter settings, and with some restructuring their grammars through resetting a number of parameters, and, in doing so, generating grammars that are neither like the L1 nor like the L2 (see Finer and Broselow, 1986 for the same argument from syntax; see also Mairs, 1989; Archibald, 1992, 1993, 1995; Özçelik, 2016, 2018 for similar findings in various domains of prosody).

These ‘stages’ do not necessarily correspond to a gradual improvement in terms of getting closer to target-like productions. In fact, they do not even necessarily parallel with increasing proficiency levels. Rather, they align with the degree to which changes have been made to the grammar. Further, these changes, as we will see, are implemented on a parameter-by-parameter basis, rather than matching with the input from the target language or certain frequency-related considerations. Still, these stages roughly correspond to the learners’ proficiency levels in that the learners with the lowest levels of proficiency were the ones who have made the fewest number of changes in their grammar, as opposed to those with the highest level of proficiency who have made the greatest number of changes. In terms of being target-like with respect to surface location of stressed or prominent syllables, however, there appears a reverse bell curve-shaped pattern in that learners seem to get worse first before getting better.

To begin with, at the first stage, there were two learners, who together comprised the group of learners with the lowest level of proficiency in Mongolian (M.I. and A.B.); these learners used L1 settings of all prosodic parameters, as would be predicted on the PAPH (or the FTFA). In other words, these learners uttered Mongolian words with English prosody, i.e., constructing right-to-left, weight-sensitive, iterative, moraic trochees where Extrametricality was set to *Yes*, and codas were moraic, as illustrated below in (18).


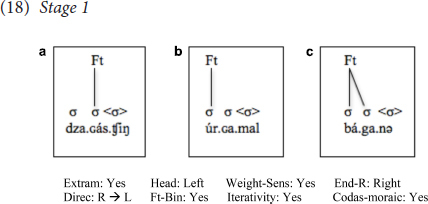


As seen, although these learners have initial stress in words that start with closed syllables, such as (18b), and cases where an open syllable is followed immediately by another open syllable, such as (18c), they fail to stress the initial syllable in all other cases, i.e., cases where the first syllable is open, and is immediately followed by a closed syllable, in which case the closed syllable is stressed, as in (18a), resulting in penultimate stress.

At the next stage were three learners (two beginners, one intermediate: T.H., P.L., K.G.) who reset Extrametricality from *Yes* to *No*, given the input illustrating many finally stressed words in Mongolian, e.g., those that contain a single long syllable which is also the final syllable in the word, as in (3). This change made their grammar less target-like on the surface regarding words with all short syllables. As illustrated in (19), for example, the learners at this stage not only fail to have initial stress in cases like (a), but also cases like (b) (for main stress) and (c), unlike the learners at the previous stage, for whom cases like (b) and (c) were still stressed on their first syllable:


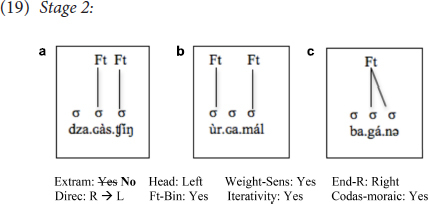


Note that the learners at this stage employ not only prosodic representations that are neither like the L1 nor the L2, but also surface stress patterns that are very much unlike both languages. Neither English nor Mongolian stresses the second syllable in cases like (19c) for example. In fact, both English and Mongolian stress the first syllable in these cases, words composed of three open/light syllables. The fact that the learners here stress the second syllable is, I argue, evidence that they make changes to their grammar *on a parameter-by-parameter basis* (see also Özçelik, 2016), which implies that they have access to these options which are made available by UG. Otherwise, we would expect them to have somewhat of a random increase in stressing the first syllable for words that are composed only of open/light syllables, and predict no intermediate stages that are otherwise inexplicable. Note also that this intermediate stage corresponds to the settings employed in certain natural languages, such as Tol (Fleming and Dennis, 1977) and Bergüner-Romansh (Kamprath, 1987), both of which employ right-to-left iterative weight-sensitive trochees with Extrametricality set to *No* (Gordon, 2014).

In addition to resetting Extrametricality from *Yes* to *No*, some learners reset End-Rule from *Right* to *Left*, as has already been mentioned earlier, a change that may have arisen to accommodate stress in words that contain two syllables with long vowels (see above). There was only one learner (intermediate: D.B) who belonged to this stage, i.e., one where *only* Extrametricality and End-Rule are reset, with no change in the values of any other parameters:


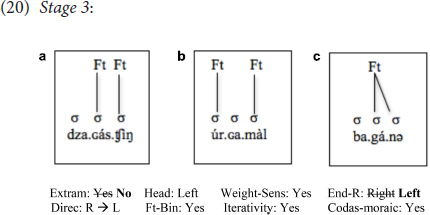


As seen, with this additional change, the learner was able to place primary stress on the initial syllable in cases like (20b) (as well as, of course, being target-like in most cases that involve heavy syllables, although those are not the subject of this section). Still, words with profiles like (20a) and (20c) did not bear initial stress. Notice, however, that this is once again a change that makes the grammar formally unlike both the L1 and L2, as End-Rule is set to *Right* in the L1, and, as the discussion in Sections “Introduction” and “Representation of Stress: the L1-L2 Language Background” demonstrates, this is most likely true for the L2, too.

In addition to the learners mentioned above who made changes to the values of Extrametricality, and, in the case of one learner, Extrametricality + End-Rule, there were four learners (three intermediate, one beginner: B.B., J.K. E.S., A.F.) who, *in addition to* resetting Extrametricality and End-Rule, reset Directionality, from *Right-to-Left* to *Left-to-Right*, consequences of which are indicated below in (21).


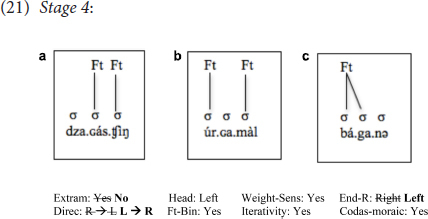


Although these learners employed completely different settings from the Stage 1 learners above who used L1 settings of all parameters [see (18)], on the surface (in terms of the location of stressed syllables), and with respect to these three word forms only, their productions looked similar, leading to the appearance of a reverse bell-shaped learning curve in stages (18) through (21), where learners start with 2 target-like forms out of 3 (18), fall all the way down to 0/3 (19) and then up to 1/3 (20) and finally up again at 2/3 (21). If one looked only at surface forms, it would, thus, have appeared to be a case of getting worse with respect to stress patterns and getting back to the starting point again, when in fact, individual grammars are being restructured along with the options made available by UG.

Finally, only two learners (K.K. and K.H.), the two most advanced among all the learners tested, had non-moraic codas, thereby treating closed syllables as light, as in the target language. One of these two learners, K.K., had reset all the parameters that were reset by the learners in the previous stage, in addition to having non-moraic codas, and thus, this change clearly put him at Stage 5:


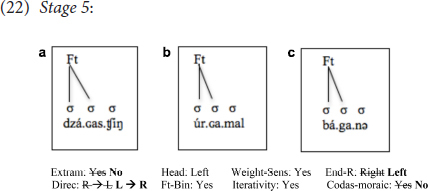


That is, with this one additional change in the value of one parameter, in addition to all other changes described above, this learner was finally able to achieve target-like representations for all Mongolian words composed only of short vowels, consistently placing stress on the first syllable of all such words, irrespective of whether certain syllables within the word end in a coda or not.

Despite being relatively target-like on the surface for words with all light syllables, this learner still had problems with words that contained multiple heavy syllables (i.e., those in Table 1), as he consistently stressed them on the leftmost syllable regardless of where the rightmost (non-final) heavy syllable was located. This is because End-Rule was set to *Left*, a fact that we understand not from his performance on stimuli with all light syllables (since codas are no longer moraic), but from those with heavy syllables. After all, looking only at his performance on stimuli with light syllables, End-Rule can either be *Left* or *Right*, as there is only one foot available (since codas are no longer moraic), and that foot is thus both the rightmost and the leftmost within the word.

The other learner who had non-moraic codas, K.H., who was also advanced, behaved slightly differently from K.K. in this regard, although the two performed similarly on stimuli with all light syllables [thus having the same representations as (18a) through (18c)]. Unlike K.K., K.H. had the (more correct) *Right* setting of the End-Rule parameter for some forms (as is again understood by her performance on stimuli with heavy syllables, like those in Table 1), see Section “Observation 2 Explained: End-Rule *Right* or *Left*, but not both or *Middle*.” It is not clear, however, whether this learner was on her way to Stage 6, in that she is starting to reset End-Rule to *Right*, after having already reset it from *Right* to *Left* (i.e., something on top of what was done by the Stage 5 learner), or that she had never reset it completely to *Left* in the first place, and kept it as *Right* for some forms, although it is not clear as to why this would be done only for the bisyllabic stimuli (see Note 4). Clearly, however, she’s treating bisyllabic stimuli differently from longer stimuli, a fact that could have something to do with her having reset Weight-Sensitivity.

## Discussion and Conclusion

In conclusion, faced with extremely complex data from the L2, and being unable to expunge the Foot from their grammar, English-speaking learners of Mongolian attained various foot-based prosodic grammars, all of which corresponded to natural languages, although most of them were neither like the L2 nor like the L1. In some cases, this led to striking patterns on the surface, such as a CV.CV.CV word bearing stress on the second syllable in the interlanguage [e.g., CV.CVì.CV, see (16c)], although both the L2 and the L1 would stress the first syllable in such cases (i.e., CVì.CV.CV), meaning that neither input from the target language nor transfer from the L1 alone could explain certain productions. In fact, even when both considerations predict the same pattern, an entirely different – and unexpected – pattern could arise. On a view where the hypothesis space is constrained by UG, such outputs are expected, as it comes as part of a change in the setting of a parameter, which results in some outputs being non-target-like, while making certain other outputs more target-like.

Just like the options the learners chose to employ, those that were not chosen were also informative, and can be analyzed under two broad categories: First, options that would not serve to make any aspect of the interlanguage more target-like on the surface were not chosen, such as the use of right-headed feet (iambs) instead of left-headed (trochees). Iambs would not have made a difference in accounting for the surface stress patterns of words with heavy syllables (i.e., rightmost stress, which can be captured equally well with trochees), while also making the default (leftmost) prominence impossible to capture, as the first syllable would (almost) never be the prominent one with iambs (unless one assumes rampant empty ON sequences for every words that begins with an open syllable. So it is the L2 input that triggers grammar restructuring, although L2 input alone is not sufficient to account for the options learners make.

Second, UG-unconstrained options, regardless of how cognitively reasonable they were, were not employed. For example, certain stages/interlanguage grammars, such as one that permits a *variable* End-Rule or End-Rule-*Middle*, did not emerge in the productions of the English-speaking subjects (despite being cognitively reasonable), again, a fact that is left inexplicable without recourse to UG, but finds a straightforward explanation on UG-based accounts, as these grammars are not permitted by the universal inventory of foot shapes (see e.g., McCarthy and Prince, 1986; Hayes, 1995). Instead, many learners reset End-Rule from *Right* to *Left*, in order to better accommodate the input, although this is not the value instantiated in the L1 and is different from the L2 setting, too. (See also Özçelik, 2016, 2018 for similar findings for Weight-Sensitivity; weight-insensitive iambs were not employed by English speakers of Turkish, as they are ruled out by the universal inventory of iamb types, although that would have been a cognitively reasonable strategy).

In addition, certain options were difficult despite being permitted by the options of UG and despite potentially leading to more target-like outputs. For example, we found that it was very difficult to change from a grammar where both long vowels and codas contribute weight to a system where only vowels do (i.e., one where only vowels are moraic, as with Mongolian), unlike the opposite direction which seems to be easy (see e.g., Archibald, 1993, 1998; see also Garcia, 2020 on the role of weight sensitivity and additional considerations, such as positional bias, that may interact with weight-sensitivity in interlanguage grammars). One reason for this could be that learners in this condition move from a superset to a subset grammar, and thus have to constrict their grammar, which is argued to be more difficult than the converse (White, 1989; Schwartz and Sprouse, 1996). Clearly, more research is needed on the role of weight sensitivity (or lack thereof) in L2 acquisition of stress systems.

An analysis of the interlanguage grammars/stages learners went through also contributes to our understanding of variability in second language phonology. Although variability in syntax and especially morphology (and, more specifically, functional morphology) has been well-investigated (see e.g., Lardiere, 1998a, b; Ionin and Wexler, 2003; White, 2003a; Ionin et al., 2004 for various proposals), variability in phonology has thus far received close to zero attention. Although well-justified prosodic approaches have been proposed in the literature, as with the Prosodic Transfer Hypothesis (see e.g., Goad et al., 2003; Goad and White, 2004, 2006, 2019), the aim was, once again, to account for morphological or syntactic variability in interlanguage grammars, not variability in phonology itself. The current research sheds light on the underlying causes of variability in interlanguage phonologies, and demonstrates how this can be captured via transfer of L1 prosodic representations and through having recourse to the options made available by UG.

Finally, note that this study was on production only; it says nothing about learners’ perception. Although it is in general good practice in L2 research to look into perception as well, there is good reason to treat stress phenomena differently, for significant perception-production asymmetries have been shown to exist for both L1 speakers, as has been demonstrated by the so called “stress deafness” phenomenon (Dupoux et al., 1997; Peperkamp and Dupoux, 2002), and for L2 speakers: those who do not correctly perceive the location of stress (with L1s that render them prone to “stress deafness”) often produce stress native-like in the L2, while those who had high perception scores behaved differently from native speakers of the target language (Altmann, 2006). Further, it is ultimately the learners’ production that renders them native- or non-native-like with regard to stress, as it is largely non-contrastive (unlike e.g., tones).

In conclusion, the current study has demonstrated that both transfer from the L1 and access to UG- seem to be relevant factors in determining the stages that learners go through (and those that they do not) in acquiring the prosody of a second language. Although it is the L2 input that triggers grammar change and restructuring, clearly, the L2 input alone is not sufficient in explaining the constructions that define interlanguage grammars or the difficulties L2 learners are faced with.

## Data Availability Statement

The raw data supporting the conclusions of this article will be made available by the authors, without undue reservation.

## Ethics Statement

The studies involving human participants were reviewed and approved by Indiana University IRB Board. The patients/participants provided their written informed consent to participate in this study.

## Author Contributions

The author confirms being the sole contributor of this work and has approved it for publication.

## Conflict of Interest

The author declares that the research was conducted in the absence of any commercial or financial relationships that could be construed as a potential conflict of interest.

## Publisher’s Note

All claims expressed in this article are solely those of the authors and do not necessarily represent those of their affiliated organizations, or those of the publisher, the editors and the reviewers. Any product that may be evaluated in this article, or claim that may be made by its manufacturer, is not guaranteed or endorsed by the publisher.

## Footnotes

1 This is crucial in that instruction or studying (or ‘learning’) the correct forms will not be of much help to the learner (more on this later), in the same way that input is not very helpful. That is, it is a unique and ideal scenario to test the involvement of UG. The question is what options learners do and do not entertain in light of extremely confusing data, no matter what the correct linguistic analysis of these data is.

2 Özçelik (2014) argues, within an Optimality Theoretical (OT) framework and focusing on Turkish, that within the same prosodic system, both End-Rule-*Left* and Leftmost-Wins could be available, and that this could capture certain cases of (otherwise random-looking) variability in the grammar. However, in the case of Mongolian, no such variability exists among speakers across words that have the same syllable structure profiles; words with similar syllable structure profiles are pronounced with the same stress pattern in Mongolian. Further, no languages have so far been identified to have a *Middle* setting for End-Rule, although End-Rule-*Left* and End-Rule-*Right* are equally possible, depending on the language (Hyman, 1977; Hayes, 1995). Similarly, although having both the *Left* and the *Right* settings of End-Rule within the same prosodic grammar is technically possible in an approach like OT, one of the two should always be predominant, and End-Rule-*Middle* should be incorrect outright, as prosodic parameters only refer to right- or left-most edges of prosodic constituents, whether the relevant constituent is the Foot, Prosodic Word or Phonological Phrase (see e.g., Nespor and Vogel, 1986; Selkirk, 1996; van der Hulst, 2014).

3 Verbs behave differently in English; only the final consonant is extrametrical for verbs, rather than the entire final syllable (Hayes, 1982).

4 This could be grammar change in progress, although that alone does not explain why the *right* setting of the parameter is used only for the bisyllabic stimuli, while the left setting is apparently used for all other stimuli, a result not predicted by our hypotheses. As one reviewer has pointed out, this could also be the effect of the data in that the surface variation leads the learner to sometimes produce initial and sometimes final stress, alternating between the two values of the parameter, perhaps until the parameter is set to its more stable value. As for why, in doing so, the learner appears to be treating bisyllabic words differently from longer words, it could have something to do with the fact that certain elements are no longer contribute weight to a syllable in her grammar, as will be explained under Stage 5 in Section “Prosodic Parameter Resetting: Stage-like Prosodic Acquisition.”
